# Re-assessment of *YAP1* and *MCR1* contributions to inhibitor tolerance in robust engineered *Saccharomyces cerevisiae* fermenting undetoxified lignocellulosic hydrolysate

**DOI:** 10.1186/s13568-014-0056-5

**Published:** 2014-07-22

**Authors:** Valeria Wallace-Salinas, Lorenzo Signori, Ying-Ying Li, Magnus Ask, Maurizio Bettiga, Danilo Porro, Johan M Thevelein, Paola Branduardi, María R Foulquié-Moreno, Marie Gorwa-Grauslund

**Affiliations:** 1 Applied Microbiology, Department of Chemistry, Lund University, P.O. Box 124, Lund, SE-22100, Sweden; 2 University of Milano Bicocca, Piazza della Scienza 2, Milan, 20126, Italy; 3 Laboratory of Molecular Cell Biology, Institute of Botany and Microbiology, Leuven, KU, Belgium; 4 Department of Molecular Microbiology, VIB, Kasteelpark Arenberg 31, Leuven, Heverlee, B-3001, Flanders, Belgium; 5 Department of Chemical and Biological Engineering, Industrial Biotechnology, Chalmers University of Technology, Gothenburg, SE-41296, Sweden

**Keywords:** Saccharomyces cerevisiae, Hydrolysate, Inhibitors, YAP1, MCR1, Ethanol

## Abstract

Development of robust yeast strains that can efficiently ferment lignocellulose-based feedstocks is one of the requirements for achieving economically feasible bioethanol production processes. With this goal, several genes have been identified as promising candidates to confer improved tolerance to *S. cerevisiae*. In most of the cases, however, the evaluation of the genetic modification was performed only in laboratory strains, that is, in strains that are known to be quite sensitive to various types of stresses. In the present study, we evaluated the effects of overexpressing genes encoding the transcription factor (*YAP1*) and the mitochondrial NADH-cytochrome b5 reductase (*MCR1*), either alone or in combination, in an already robust and xylose-consuming industrial strain of *S. cerevisiae* and evaluated the effect during the fermentation of undiluted and undetoxified spruce hydrolysate. Overexpression of either gene resulted in faster hexose catabolism, but no cumulative effect was observed with the simultaneous overexpression. The improved phenotype of *MCR1* overexpression appeared to be related, at least in part, to a faster furaldehyde reduction capacity, indicating that this reductase may have a wider substrate range than previously reported. Unexpectedly a decreased xylose fermentation rate was also observed in *YAP1* overexpressing strains and possible reasons behind this phenotype are discussed.

## Introduction

Production of second-generation bioethanol from lignocellulosic biomass requires robust *Saccharomyces cerevisiae* strains with improved capacity to cope with the toxic compounds formed during the biomass pre-treatment, among which are 5-hydroxymethylfurfural (HMF), furfural, weak organic acids and phenolic compounds (Parawira and Tekere [2011]). This has led to extensive studies to decipher mechanisms behind the compounds toxicity and the yeast natural tolerance responses to them and, among others, genes involved in detoxification and yeast tolerance to individual inhibitors have been identified, such as *ADH6, HAA1* or *PMA1* (Haitani et al. [2012]; Mira et al. [2010]; Petersson et al. [2006]); for a more exhaustive review, see (Almeida et al. [2009a]; Liu [2011]). *YAP1* is another interesting candidate for industrial strain engineering because it encodes a transcription factor (Yap1p) that simultaneously controls a wide range of stress-related targets (Toone and Jones [1999]). Notably, its overexpression has a beneficial role in the response of laboratory *S. cerevisiae* towards HMF, furfural, and different concentrations of hydrolysate (Alriksson et al. [2010]; Kim and Hahn [2013]; Ma and Liu [2010]; Sundström et al. [2010]). Another interesting and complementary candidate for gene overexpression is *MCR1* that encodes the mitochondrial NADH-cytochrome b5 reductase (Hahne et al. [1994]; Meineke et al. [2008]). Previous experiments performed in our group revealed that overexpression of *MCR1* in *S. cerevisiae* resulted in a reduced lag phase and faster growth rate when the yeast was grown with high concentrations of acetic acid (Signori et al., personal communication). This weak acid is one of the inhibitors directly affecting xylose metabolism in *S. cerevisiae* (Almeida et al. [2011]; Bellissimi et al. [2009]; Casey et al. [2010]; Helle et al. [2003]). Still, considering that many of the studies about strain improvement towards hydrolysate-derived inhibitors concern laboratory strains, it is difficult to predict the real effect of these changes in an industrial, and more robust, strain background.

The objective of the present study was to evaluate the effect of overexpressing *YAP1* and *MCR1*, either alone or in combination, in process-like conditions, that is using a robust industrial *S. cerevisiae* strain and undetoxified lignocellulosic hydrolysate. For this, the strain GSE16 was chosen as background strain for engineering since it combines a robust industrial background with the ability to ferment xylose, using the xylose isomerase pathway (Demeke et al. [2013b]). This robust strain was developed by a combination of different strategies including rational metabolic engineering, mutagenesis, evolutionary engineering, genome shuffling and meiotic recombination (Demeke et al. [2013a],[b]). Moreover, and as part of the genetic engineering strategy used in the current study, the overexpression of *YAP1* was combined with the deletion of the chaperone-encoding gene *APJ1* since deletion of this gene has been previously reported to enable growth on rich medium at inhibitory ethanol concentrations for the parental strain (Swinnen et al. [2012]). The relevance of overexpressing *YAP1* and *MCR1* in an industrial background was confirmed for undetoxified spruce hydrolysate fermentation. We also uncovered unexpected interactions between *YAP1* overexpression and xylose metabolism.

## Materials and methods

### Strains

*S. cerevisiae* strains utilized in this study are presented in Table 1. *Escherichia coli* DH5α and *E. coli* NEB 5-alpha were used for sub-cloning and were grown on Luria-Bertani (LB) medium supplemented with 100 mg.L^−1^ ampicillin, when required. Plasmids utilized in the study are described in Table 2.

**Table 1:** ***Saccharomyces cerevisiae*** **strains used in the current work**

***S. cerevisiae*** **strains**	**Genotype**	**Plasmid**	**Source**
CEN.PK 102-5b	*Mat a, ura 3–52, his2∆1, leu 2-3/112*		(van Dijken et al. [2000])
CEN.PKc	CEN.PK 102-5b	[pYX012; pYX022; pYX042]	This work
TMB3400	*S. cerevisiae* industrial strain		(Almeida et al. [2009b])
GSE16	GS1.11-26 + backcrossing with a segregant of Ethanol Red that is tolerant towards acetic acid; *MAT*α*/*α		(Demeke et al. [2013b])
GSE16 - YAP1	GSE16*-APJ1*-1:: *TDH3*p*-YAP1-CYC1*t		This work
GSE16 - MCR1	GSE16*- YLR446W-1*::*TPI*p-MCR1		This work
GSE16 - MCR1-YAP1	GSE16- *YLR446W-1*::*TPI*p-MCR1*, APJ1*-1:: *TDH3*p*-YAP1-CYC1*t		This work
GSE16 - ΔΔAPJ1	GSE16-APJ1/APJ1::attL/attL		This work

**Table 2:** Plasmids used in the current work

**Plasmids**	**Relevant features**	**Origin**
p426GPD	*Multicopy URA3* 2 μm *TDH3*p-*CYC1*t	(Mumberg et al. [1995])
p426GPD –YAP1	*TDH3*p*-YAP1-CYC1*t	This work
pUG6	*kanMX* flanked by *loxP* sites	(Güldener et al. [1996]) (EUROSCARF; accession number P30114)
YE-plac 112 KanR	Multicopy*,* KanMX	(Jeppsson et al. [2003])
YE-plac 112 KanR-YAP1	*TDH3*p*-YAP1-CYC1*t	This work
pJET1,2	Multicopy	Thermo Scientific, Belgium
pJET1,2-attB-KanMX-attP	Multicopy, KanMX under TEFp	This work
p-intYAP1	*TDH3*p*-YAP1-CYC1*t *–* KanMX attB/attP system; integrative	This work
pSTBlue-1	multi-purpose cloning vector with dual kanamycin/ampicillin resistance.	Novagen (EMD Millipore)
pSTBlue-YLR446W	pSTBlue-1 with the *YLR446W* gene cloned into the multiple cloning region	This work
pYX012	Integrative; *URA3*; TPI1p	R&D System, Inc., Wiesbaden, D
pYX012-LoxPkanMXLoxP	pYX012, with the KanMX cassette flanked by loxP sites, deriving from pUG6	This work
pYX012-LoxPKanMXLoxP-MCR1	pYX012- LoxPKanMXLoxP with the *MCR1* gene inserted in the MCS under the control of the TPI1 promoter	This work
pSTBlue-YLR446WΔ- LoxPKanMXLoxP-MCR1	pSTBlue-YLR446W with the LoxPKanMXLoxP-MCR1 cassette, deriving from pYX012 LoxPKanMXLoxP-MCR1, inserted into the YLR446W sequence	This work
pSH65	Centromeric plasmid, GAL1p-cre, ble^r^	(Gueldener et al. [2002]) (EUROSCARF; accession number P30122)
pJET1,2- attB-hph- attP	Multicopy, hph under TEFp	This work
pBEVY-Nat-phiC31	Multicopy, PhiC31 integrase	This work

### Molecular biology methods

Standard molecular biology methods were used for all cloning procedures. (Sambrook and Russel [2001]). ThermoScientific GeneJET plasmid miniprep kit (ThermoScientific, Lithuania) was used for plasmid extraction. E.Z.N.A CyclePure Kit (Omega Biotek, USA) was used for purification of polymerase chain reaction (PCR) products. Qiagen Qiaquick gel extraction kit (Qiagen GmbH, Germany) was used to extract DNA from agarose gels. All DNA-modifying enzymes were purchased from ThermoScientific. Primers for PCR and sequencing of DNA constructs were ordered from and performed by MWG (MWG-Biotech AG, Germany). All primers are shown in Additional file 1: Table S1. Transformation of *E. coli* was performed using the Inoue Method (Sambrook and Russel [2001]). Either the lithium acetate (LiAc) method (Gietz et al. [1995]), a modified version of it that uses dimethyl sulfoxide (DMSO) (Hill et al. [1991]) or electroporation (Benatuil et al. [2010]), were used as transformation methods of *S. cerevisiae*.

### Construction of plasmids pJET1,2-attB-KanMX-attP, pJET1,2-attB-hph-attP and pBEVY-Nat-PhiC31

The antibiotic markers KanMX and hph expressed under the TEF promoter and terminator were amplified with the primers Fw-A1-attP-tefpr and Rv-A2-attB-teft and cloned into pJET1,2 (Thermo Scientific, Belgium) following the protocol of the kit. The resulting plasmids were called pJET1,2-attB-KanMX-attP and pJET1,2-attB-hph-attP respectively. The PhiC31 integrase was amplified from pCMVInt (Addgene, Cambridge, Massachusetts, USA) using the primers Fw-PstI-PhiC31 and Rv-BamHI-PhiC31 and cloned into the *Pst*I and *Bam*HI sites of pBEVY-Nat giving the pBEVY-Nat-PhiC31.

### Construction of S. cerevisiae strains

#### *S. cerevisiae* GSE16-YAP1

The open reading frame of the *YAP1* gene was amplified from *S. cerevisiae* strain TMB3500 (Almeida et al. [2009b]), using primers YAP1-F and YAP1-R. The amplicon was ligated into p426GPD (Mumberg et al. [1995]) resulting in p426GPD-YAP1 used for transformation of *E. coli* DH5α cells and followed by sequence verification. The cassette from p426GPD-YAP1 was amplified using primers GPD-YAP1-F and CYC1t-YAP1-R. The amplified cassette was ligated into pUG6 after restriction with *Aat*II and *Pvu*II, resulting in pUG6-YAP1. Amplification of the two homologous regions (HR) used for integration of the *YAP1* cassette into the *APJ1* locus were performed from *S. cerevisiae* GSE16 (Demeke et al. [2013b]) with primers HR1-F/HR1-R and HR2-F/HR2-R. Amplicon for RH2 was first ligated into pUG6-YAP1 and transformed into *E. coli* DH5α cells resulting in pYAP1-HR2. Amplicon for HR1 was ligated into pYAP1-HR2 and transformed into *E. coli* DH5α cells resulting in pYAP1-HR2-HR1. The selection cassette (KanMX) flanked by the attB and attP sites was amplified from pJET1,2-attB-KanMX-attP using attBP-F and attBP-R. The amplicon was ligated into pYAP1-HR2-HR1 and transformed into *E. coli* NEB 5-alpha resulting in p-intYAP1. The nucleotide sequence of each amplicon was verified after every subsequent cloning step. *S. cerevisiae* strain GSE16 was used for expression of the *YAP1* construct. In this strain, the expression cassette containing the transcription factor *YAP1* was integrated by linearization of the integrative cassette using *Aat*II and *Not*I, followed by transformation using a DMSO-modified version of the LiAc method (Hill et al. [1991]). The selection of colonies was done on YNB plates with 150 μg.mL^−1^ geneticin G418 (Sigma). Verification of the correct insertion (*APJ1* locus) was done by sequencing using primers Ver.ins1-F/Ver.ins1-R and Ver.ins2-F/Ver.ins2-R. The resulting strain was named GSE16-YAP1.

#### *S. cerevisiae* GSE16-MCR1

The open reading frame of *MCR1* was amplified from CEN.PK 102-5b using primers MCR1-F and MCR1-R. The amplified DNA fragment (1544 bp) was cloned into pSTBlue (*Eco*RV site) and used to transform *E. coli* DH5 α cells. A 1.5 Kb *Eco*RI fragment containing *MCR1* was isolated from pSTBlue-MCR1 and cloned into the MCS of the yeast integrative plasmid pYX012, resulting in pYX012-MCR1. In parallel, the ORF YLR446W was amplified from *S. cerevisiae* CEN.PK 102-5b using primers YLR446W-F and YLR446W-R and cloned into pSTBlue (*Eco*RV site) resulting in pSTBlue-YLR446W. The selection cassette (KanMX) flanked by two LoxP sites was amplified from pUG6 using primers YLR446W Lox-F and Lox-R, and cloned into pYX012-MCR1 plasmid (*Kpn*I site). The expression cassette (LoxPKanMXLoxP + promoter + *MCR1* ORF + terminator) was amplified using primers YLR446W Lox-F and YLR446W TER-R and cloned into pSTBlue-YLR446W after restriction with *Btg*I and *Bsr*GI (this double digestion allowed the removal of the inner part of the *YLR446W* gene (~601 bp)). Each amplicon was verified by sequencing analysis after every subsequent cloning step. *S. cerevisiae* strain GSE16 was used for expression of the *MCR*1 construct. The expression cassette containing *MCR1* was integrated into GSE16 after PCR amplification using primers YLR446W-F and YLR446W-R. Transformation was carried out using a DMSO-modified version of the LiAc method (Hill et al. [1991]). Correct insertion in the *YLR446W* locus was verified by PCR. The resulting strain was named GSE16-MCR1. The removal of the dominant marker (KanMX) was obtained by transforming GSE16-MCR1 with pSH65.

#### *S. cerevisiae* GSE16-MCR1-YAP1

The strain was constructed from GSE16-MCR1 by integration of p-intYAP1 previously digested with *Aat*II and *Not*I (see *S. cerevisiae* strain overexpressing *YAP1*). The correct integration in the *APJ1* locus was verified by PCR.

#### *S. cerevisiae* GSE16- ΔΔAPJ1

The selection cassettes (KanMX and hph) flanked by the attB and attP sites were amplified by PCR from pJET1,2-attB-KanMX-attP and pJET1,2- attB-hph- attP using primers Fw-APJ1-A1 and Rv-APJ1-A2 with 50 bp homologues regions. Deletion of the two *APJ1* alleles was carried out by integrating both selection cassettes into the *APJ1* loci of GSE16 (Demeke et al. [2013b]) using an adapted electroporation method (Benatuil et al. [2010]). *APJ1* double deletion colonies were selected from YPD plates with hygromycin (300 μg.mL^−1^) and geneticin (G418) (200 μg.mL^−1^), and checked by PCR using the primers Fw-APJ1-check and Rv-APJ1-check. To loop out the markers, the colonies were transformed with pBevy-Nat-phi31 and selected in YPD plates with 100 μg.mL^−1^ of nourseotricin. These colonies were also checked on YPD hygromycin and YPD geneticin plates. The plasmids containing the integrase were lost by growing the colonies in YPD liquid medium overnight and transferred twice. The resulting strain was named GSE16-ΔΔAPJ1.

##### Spruce hydrolysate

Spruce hydrolysate was obtained from SEKAB E-Technology AB (Örnsköldsvik, Sweden), and consisted of the non-detoxified liquid fraction of spruce after a pretreatment by SO_2_ catalyzed steam explosion. It is referred in this work as spruce hydrolysate and had the following sugar composition: 11 g.L^−1^ glucose, 17 g.L^−1^ mannose, 4 g.L^−1^ galactose, and 10 g.L^−1^ xylose. All the batch fermentations were carried out with the same batch of spruce hydrolysate which was kept at 4°C.

##### Anaerobic batch fermentations of spruce hydrolysate

Inoculum was prepared by growing the cells overnight in 1 L shake flasks containing 100 mL defined mineral medium with vitamins (Verduyn et al. [1992]) with glucose as carbon source (20 g.L^−1^) buffered with phthalate buffer (50 mM, pH 5.0). After centrifugation and a washing step with deionized water, the pellet was resuspended with 20 mL of fermentation medium, and immediately used for inoculation of the fermenter. The fermentation medium consisted of 100% (v/v) spruce hydrolysate supplemented with 10 g.L^−1^ of xylose, 1 g.L^−1^ yeast extract, 0.5 g.L^−1^ (NH_4_)_2_HPO_4_ and 0.025 g.L^−1^ MgSO_4_. 7H_2_O. The pH of the hydrolysate was adjusted to 5.0 with 8 M KOH prior to supplementation. The fermentations were carried out in 1.2 L MultiFors fermenters with 0.5 L working volume. Temperature was maintained at 30°C and the pH was kept at 5.0 by addition of 3 M KOH. Oxygen free conditions were maintained by sparging N_2_ at 0.2 L.min^−1^, and the agitation was set to 600 rpm. Reactors were inoculated to a biomass concentration of 1 g.L^−1^ (cdw). Cultivations were performed in biological duplicates for each investigated strain. Specific conversion rates of furfural and HMF were calculated assuming a pseudo-steady state during the exponential growth on glucose.

##### Anaerobic batch fermentations in mineral medium

Inoculum was prepared by growing the cells overnight in 100 mL shake flasks containing 20 mL defined mineral medium with vitamins (Verduyn et al. [1992]) with glucose as carbon source (20 g.L^−1^) buffered with phthalate buffer (50 mM, pH 5.0). Fermentations were carried out in 100 mL glass bottles with 40 mL working volume of the same mineral medium and vitamins supplemented with 5 g.L^−1^ of glucose and 20 g.L^−1^ of xylose as carbon and energy sources. Temperature was maintained at 30°C and stirring was set at ca. 160 rpm. Oxygen-limited conditions were obtained by sealing the bottles and sparging N_2_ at 0.2 L.min^−1^ for at least 5 minutes before inoculation. A cotton-filled syringe was inserted through the rubber stopper using a needle to avoid accumulation of gas inside the bottles. Bottles were inoculated to a biomass concentration of ca. 1 g.L^−1^ (cdw). Cultivations were performed in biological duplicates for each investigated strain.

##### Analysis of substrates and products

Controlled volumes of samples were taken regularly for analysis. Biomass was followed by OD620 measurements during the length of the fermentations and determination of cell dry weight measurements were also done at time zero (just after inoculation) and at times 42 h and 92 h. For biomass determination, the cell pellet from 5 mL culture was washed with distilled water and dried on Gelman filters (ø 47 mm Supor-450, 0.45 μm) in a microwave oven (350 W) for 8 minutes. Ethanol, glycerol, acetic acid, HMF and furfural were analysed by high performance liquid chromatography (HPLC; Waters Corporation, MA, USA) using an Aminex HPX-87H column (Bio-Rad, CA, USA) at 65°C. The mobile phase was 5 mM sulphuric acid with a flow rate of 0.6 mL.min^−1^. Analysis of glucose, mannose, xylose, galactose and xylitol was performed on a Shodex™ SP-0810 sugar column (Showa Denko K.K, Japan) at 85°C with water as mobile phase and 0.6 mL.min^−1^ flow rate. All compounds were detected with a refractive index detector (Shimadzu, Tokyo, Japan, and Waters 2414, MA, USA respectively). Yields were calculated based on HPLC measurements.

## Results

### Genetic engineering of the xylose consuming industrial strain GSE16

It has previously been reported that, whereas overexpression of the transcription factor *YAP1* resulted in a strain with a faster sugar consumption rate when fermenting 60% (v/v) of spruce hydrolysate, the same strain was severely inhibited at higher concentrations of the substrate (Alriksson et al. [2010]). However the study was performed using laboratory strains with a sensitive genetic background towards hydrolysate-derived inhibitors (Martin and Jönsson [2003]). From these results and further studies carried out in our group (data not shown), the importance of the genetic background and the initial inhibitors concentrations on the effect of *YAP1* overexpression was further highlighted, and stressed the necessity of assessing the effect of gene modification under more industrial relevant conditions, that is using up-to-date engineered industrial strains and process-like fermentation conditions.

In this study, overexpression cassettes containing the genes that encode the transcription factor *YAP1* and the mitochondrial NADH-cytochrome b5 reductase *MCR1* were designed and integrated in the genome of the robust industrial strain GSE16, either alone or in combination, generating strains GSE16-YAP1, GSE16-MCR1 and GSE16-YAP1-MCR1 (Table 1). Simultaneous *YAP1* integration and *APJ1* deletion were obtained by inserting the *YAP1* overexpression cassette into the *APJ1* locus. The *MCR1* cassette was targeted to the YLR446W locus, a gene whose deletion has not affected the yeast performance during fermentation (Subtil and Boles [2012]). A control strain in which both alleles of *APJ1* were deleted (GSE16-ΔΔAPJ1) was included during the fermentations (Table 1).

The effect of each genetic modification was assessed during anaerobic batch fermentations of undiluted spruce hydrolysate. In order to have a medium composition similar to large scale lignocellulose-based fermentations, spruce hydrolysate with limited nutrient supplementation was used (See Materials and methods). The xylose concentration was increased from 10 g.L^−1^ to 20 g.L^−1^ to allow a longer period for analysis of the xylose consumption phase. The initial composition of the fermentation medium consisted therefore of 11 g.L^−1^ glucose, 17 g.L^−1^ mannose, 4 g.L^−1^ galactose, and 20 g.L^−1^ xylose. The following inhibitors could also be identified: acetate (3.7 g.L^−1^), HMF (0.96 g.L^−1^) and furfural (0.78 g.L^−1^). The strain robustness was first assessed by comparing lag phase duration, specific growth rate, and specific HMF and furfural conversion rates. Next, we evaluated the fermentation performance of the different strains in terms of glucose and xylose consumption rates, ethanol production rate, and product distribution. The carbon dioxide profile of all strains during the batch fermentations is presented in Additional file 1: Figure S1.

### Impact of strain engineering on growth and furaldehyde conversion

During the anaerobic batch fermentations of 100% spruce hydrolysate, and with an initial biomass of 1 g.L^−1^cell dry weight (cdw), the control strain GSE16 showed a lag phase of 9 hours (Table 3). The strains overexpressing *YAP1* showed a consistent decrease in the duration of the lag phase, which lasted for 5.3 ± 0.6 hours. The lag phase duration was not significantly affected by overexpression of *MCR1* while it was increased (by ~3 h) for the strain ΔΔAPJ1 (p =0.01). The specific growth rate was also altered in all the strains with the exception of ΔΔAPJ1. GSE16-YAP1 showed a specific growth rate of 0.21 h^−1^, that is an increase of around 60% when compared to the control strain (p = 0.08). A similar increase was also displayed by GSE16-MCR1 (p = 0.15) (Table 3). No additional increase was observed by the strain combining both modifications.

**Table 3:** Lag phase duration and maximum specific growth rate during batch fermentation of spruce hydrolysate

**Strain**	**Lag phase**^**a**^**(h)**	**Growth rate, μ **^**b**^**(h**^**−1**^**)**
**GSE16**	8.96 ± 0.08	0.13 ± 0.02
**GSE16 – YAP1**	5.29 ± 0.59	0.21 ± 0.03
**GSE16 – MCR1**	7.65 ± 1.06	0.20 ± 0.04
**GSE16 – YAP1- MCR1**	5.49 ± 0.83	0.21 ± 0.02
**GSE16 –** ΔΔ**APJ1**	12.04 ± 0.48	0.12 ± 0.02

Initial biomass concentration was 1 g.L^−1^ cdw. The values show the mean and standard deviation of two biological replicates.

^a^The lag phase is defined as the time between inoculation and the onset of the increase on carbon dioxide production. ^b^Growth rates were calculated from the carbon dioxide production rates during the exponential phase on glucose.

**Figure 1:**
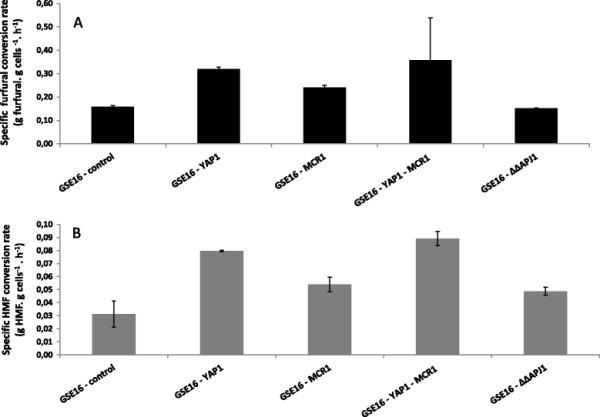
***In vivo *specific furfural (A) and HMF (B) conversion rates (g.g cells**^**−1**^ **h**^**−1**^**) displayed by the different strains during fermentation of spruce hydrolysate.** The values were calculated during the first two hours (furfural) and ten hours (HMF), considering the initial cdw (1 g.L^−1^). The bars represent mean values of two biological duplicates, and the error bars indicate the standard deviation.

As for furaldehyde conversion, all strains exhibited a higher specific conversion rate (g.g cells^−1^.h^−1^) for furfural than for HMF (Figure 1). The control strain displayed a furfural specific conversion rate of 0.16 g.g cells^−1^.h^−1^. GSE16-YAP1 showed the highest furfural specific conversion rate among all the strains (two times higher than the control strain, p = 0.002). Conversion of furfural was also improved when *MCR1* was overexpressed (Figure 1). Likewise, in vivo conversion of HMF was enhanced by overexpression of *YAP1* or *MCR1*. GSE16-YAP1 was able to convert the inhibitor around two times faster (0.08 g.g cells^−1^.h^−1^) than the parental strain (p = 0.02), and a 67% increase (p = 0.11) was observed for GSE16-MCR1 (Figure 1). As observed for the growth rate, overexpression of *YAP1* in combination with *MCR1* did not result in additional improvements neither in furfural nor in HMF conversion capacity. Neither was any significant difference in reduction capacity observed between the control strain and the *APJ1* double deletion mutant.

### Impact of strain engineering on sugar metabolism

Cell dry weight (cdw) measured after 48 and 92 h of fermentation, suggested that all strains had a similar increase in biomass (data not shown). However, accurate determination of biomass concentration in the presence of lignocellulosic hydrolysate was made difficult by the presence of solid particles in suspension in the medium or because of variations in the broth color (in the case of optical density measurement) over time. Therefore the strains were compared in terms of volumetric rates (of sugar and product formation) instead of specific rates.

The volumetric consumption rate of glucose was around 0.81 g.L^−1^.h^−1^ for the strains overexpressing *YAP1* or *MCR1*, which was at least 65% higher than the rate observed for the control strain (Table 4). And again, no additive effect was seen in the strains combining the overexpression of both genes. Deletion of *APJ1* resulted in a marginal increase in glucose utilization rate. Our analytical set-up did not allow accurate quantification of the mannose and galactose consumption rates. However, approximate determinations showed that mannose was consumed shortly after glucose was depleted from the medium. Co-consumption of mannose and xylose was observed during this phase. After mannose was exhausted from the medium, the relatively low amount of galactose (4 g.L^−1^) was co-consumed together with xylose (data not shown). In contrast to glucose, the xylose consumption rate was found to be negatively affected by *YAP1* overexpression. While the control strain consumed 0.22 g xylose.L^−1^.h^−1^, a 40% reduction was observed for the two strains overexpressing the transcription factor (p ≤ 0.03). Overexpression of *MCR1* alone and deletion of *APJ1* did not have a significant effect on xylose utilization (Table 4). Since the deletion of both alleles of *APJ1* in GSE16-ΔΔAPJ1 did not affect xylose consumption, it is very unlikely that the effect observed in the *YAP1*-overexpressing strains (carrying only one deleted allele) could be responsible for the lower xylose consumption rate observed in these strains.

**Table 4:** Volumetric consumption rate of glucose and xylose and volumetric production rate of ethanol during fermentation of spruce hydrolysate

**Strain**	**Volumetric consumption and production rates (g.L**^**−1**^**.h**^**−1**^**)**				
	**Glucose**	**Xylose**	**Ethanol (glucose phase)**	**Ethanol (xylose phase**^**+**^**)**	**Ethanol (overall)**
**GSE16**	0.49 ± 0.01	0.22 ± 0.01	0.60 ± 0.13	0.13 ± 0.00	0.27 ± 0.04
**GSE16 – YAP1**	0.81 ± 0.15	0.13 ± 0.00	0.90 ± 0.00	0.09 ± 0.02	0.21 ± 0.00
**GSE16 – MCR1**	0.81 ± 0.12	0.24 ± 0.02	0.84 ± 0.08	0.13 ± 0.00	0.27 ± 0.01
**GSE16 – YAP1- MCR1**	0.84 ± 0.12	0.13 ± 0.00	0.90 ± 0.08	0.08 ± 0.00	0.20 ± 0.01
**GSE16 –** ΔΔ**APJ1**	0.64 ± 0.03	0.19 ± 0.02	0.57 ± 0.05	0.11 ± 0.01	0.28 ± 0.01

^+^During this phase galactose is also consumed.

The volumetric ethanol production rate was calculated both during the glucose and the xylose consumption phases (Table 4). As expected from the differences between the rates of consumption of the two sugars, the ethanol production rate measured during the xylose phase was considerably lower than the one observed during the glucose phase for all strains. For the control strain, for example, the ethanol production rate on xylose decreased by 78% as compared to the one measured during the glucose phase. When comparing the different strains on glucose, GSE16-YAP1 showed one of the fastest ethanol production rates (0.90 g ethanol.L^−1^.h^−1^, p = 0.08). More generally, the ethanol production rate calculated for the new constructs was between 18% and 53% higher than the one obtained for the control strain, thereby matching the increase in the glucose consumption rate. An exception to this correlation was GSE16- ΔΔAPJ1, for which the ethanol rate during the glucose phase was the same as for the control strain (Table 4). On xylose, both *YAP1*- overexpressing strains exhibited a 40% reduction in the ethanol production rate when compared to GSE16. For GSE16-MCR1 and GSE16- ΔΔAPJ1, the ethanol rate in the xylose phase was comparable with that of the control strain. When considering the total length of the fermentations (92 h), no substantial differences were observed between the strains in terms of the volumetric production rate of ethanol (p>0.1) (Table 4).

All strains showed similar metabolite distributions, with the exception of xylitol which was around 2.4 times higher for the strains overexpressing *YAP1* (p ≤ 0.03) (Table 5). The ethanol yields at the end of the fermentation were very similar between all strains, and accounted for about 50-58% of the maximum theoretical yield (Table 5). These low ethanol yields are very likely a result of a significant rate of evaporation in the fermenter. As previously reported (Bengtsson et al. [2009]; Wahlbom et al. [2001]), the low boiling point of ethanol and the continuous sparging of the fermenters with nitrogen importantly affect the ethanol yields during prolonged fermentations. As mentioned earlier, the biomass yield was also similar for all strains as was the glycerol yield. The acetate yield was very low for all the strains and less than 0.01 g/g in all the cases (data not shown).

**Table 5:** Ethanol, glycerol, biomass and xylitol yields per gram of consumed sugars in anaerobic batch fermentation of spruce hydrolysate

**Strain**	**Yields**^**1**^**(g.g**^**−1**^**)**			
	**Y**_**ethanol**_	**Y**_**glycerol**_	**Y**_**biomass**_	**Y**_**xylitol**_
**GSE16**	0.27 ± 0.08	0.05 ± 0.00	0.04 ± 0.01	0.05 ± 0.01
**GSE16 – YAP1**	0.28 ± 0.05	0.05 ± 0.01	0.04 ± 0.00	0.12 ± 0.00
**GSE16 – MCR1**	0.26 ± 0.00	0.06 ± 0.00	0.04 ± 0.00	0.05 ± 0.01
**GSE16 – YAP1- MCR1**	0.25 ± 0.00	0.06 ± 0.00	0.04 ± 0.00	0.12 ± 0.01
**GSE16 –** ΔΔ**APJ1**	0.30 ± 0.00	0.04 ± 0.00	0.04 ± 0.00	0.07 ± 0.02

Yields of ethanol, glycerol and biomass were calculated based on the total consumed sugars, while xylitol yields were calculated based on the consumed xylose only. The numbers reported are means ± standard deviation (n = 2).

In summary, the deletion of *APJ1* in GSE16 was not detrimental for the strain performance when fermenting spruce hydrolysate. While it was not possible to evaluate the positive benefit of this deletion with the set-up used for the fermentations (expected concentrations of ethanol lower than 3% v/v), *APJ1* can be considered as a good candidate for integration of expression cassettes in strains to be used during very high gravity fermentations. In contrast, *YAP1* and *MCR1* had a positive effect on glucose fermentation in undetoxified hydrolysate but the effect was not cumulative. Finally, *YAP1* overexpression had an unexpected negative impact on xylose utilization.

### YAP1 and xylose consumption in mineral medium

**Figure 2:**
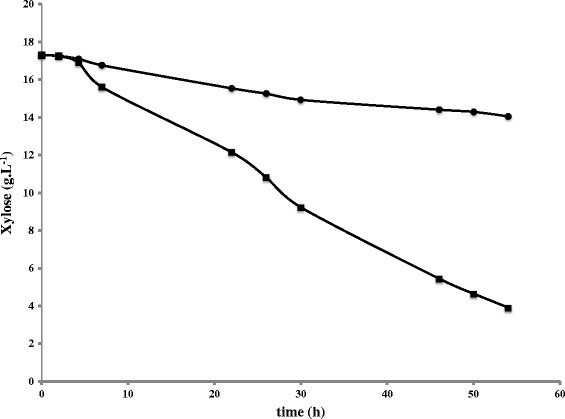
**Profile of xylose consumption for GSE16 (squares) and GSE16-YAP1 (circles) in small vials with mineral medium and 5 g.L**^**−1**^**glucose + 20 g.L**^**−1**^**xylose.** Initial biomass was 1 g.L^−1^ cdw. The experiment was carried out in biological duplicates. The figure shows the data of a representative profile for each strain with deviation <10%.

The positive effect of *YAP1* in relation to resistance to inhibitors was clearly shown by the improved fermentation rate of glucose during the anaerobic batch fermentations of spruce hydrolysate. The effect during the xylose phase, on the other hand, was not clear. In order to further investigate the effect of *YAP1* overexpression on xylose consumption and to prevent any interference caused by the complex matrix of spruce hydrolysate, the xylose consumption rate of strain GSE16-YAP1 was evaluated during anaerobic fermentation of mineral medium containing 5 g.L^−1^ of glucose and 20 g.L^−1^ xylose. Strain GSE16 was included as a control. With an initial biomass of 1 g.L^−1^ (cdw), both strains consumed all the glucose within 5 hours. However, the negative effect of *YAP1* overexpression on xylose utilization was also observed in mineral medium, indicating that such effect was not related to the presence of inhibitors. After 54 h, the control strain GSE16 consumed 13.06 ± 0.46 g of xylose, while GSE16-YAP1 only consumed 3.17 ± 0.1 g xylose (Figure 2). As observed during the fermentations of hydrolysate, the final yields of biomass and extracellular metabolites were similar between the strains, except for xylitol. After 138 h, the xylitol yield of GSE16-YAP1 was 93% higher than that of the control strain (Table 6).

**Table 6:** **Ethanol, glycerol, biomass, xylitol and acetate yields obtained during fermentations on mineral medium with 5 g.L**^**−1**^**glucose and 20 g.L**^**−1**^**xylose**

**Strain**	**Yield (g.g**^**−1**^**)**				
	**Y**_**Ethanol**_	**Y**_**Glycerol**_	**Y**_**Biomass**_	**Y**_**Xylitol**_	**Y**_**Acetate**_
GSE16	0.45 ± 0.03	0.04 ± 0.00	0.09 ± 0.00	0.03 ± 0.00	0.01 ± 0.00
GSE16-YAP1	0.45 ± 0.02	0.04 ± 0.00	0.10 ± 0.00	0.06 ± 0.00	0.01 ± 0.00

Yields of ethanol, glycerol, biomass and acetate were calculated based on the total sugar consumed, while xylitol yields were calculated based on the consumed xylose only. The numbers reported are means ± standard deviation (n = 2).

## Discussion

In the present study two different genetic modifications that have been reported to improve strain tolerance in a laboratory strain background were introduced in the industrial *S. cerevisiae* strain GSE16 and assessed during fermentation of undetoxified spruce hydrolysate. The objective was to test whether the overexpression of the selected genes would still be relevant in a strain with a high robust genetic background, to identify the most promising gene among the two candidates, and to assess any putative additive or synergistic effects.

During the glucose consumption phase, the overexpression of *YAP1* was shown to be relevant for inhibitor conversion, even in a robust genetic background. Similarly, the strain robustness in relation to spruce inhibitors was further increased when overexpressing *MCR1,* but no cumulative effect of combined overexpression was revealed. Slightly increased inhibitor tolerance was observed when overexpressing *YAP1* as compared to *MCR1*, which might be explained by the diversity of genes controlled by this transcription factor with relevant functions for detoxification (reductases, transporters, and oxidative stress-related enzymes among others) (Alriksson et al. [2010]; Herrero et al. [2008]; Toone and Jones [1999]). Still, the explanation for the almost equivalent effect obtained by *MCR1* overexpression is less evident. Previous results showed that overexpression of *MCR1* resulted in better growth in mineral medium supplemented with 12 g.L^−1^ acetic acid. (Signori et al., personal communication). Considering the role of the enzyme encoded by *MCR1* in maintaining the antioxidant D-erythroascorbic acid (EASC) in its reduced form (Lee et al. [2001]), it is plausible that higher concentrations of this antioxidant may help counteracting the oxidative effect exerted by the acetic acid present in the hydrolysate (Semchyshyn et al. [2011]). In fact, the effect seen by *MCR1* overexpression in the presence of acetic acid could be similar to the effect obtained with the biosynthesis of ascorbic acid (ASC) reported by Branduardi (Branduardi et al. [2007]). In this study the authors showed that in yeasts, biosynthesis of ASC -a molecule very similar in structure and properties to D-erythroascorbic acid (EASC) - conferred increased resistance to H_2_O_2_, low pH and organic acids. Moreover, the higher in vivo conversion rates of furaldehydes displayed by GSE16-MCR1 also points towards an involvement of the enzyme in the reduction (by a yet unknown mechanism) of HMF and furfural probably into their less inhibitory alcohol forms (Liu et al. [2005]). Given the specific location of this enzyme, this result supports previous observations that indicated a damaging effect of hydrolysate-derived inhibitors to the mitochondria (Allen et al. [2010]; Nguyen et al. [2014]). To the best of our knowledge this is the first report of a positive effect of *MCR1* overexpression on hydrolysate detoxification; and all together the results suggest that increased concentrations of NADH-cytochrome b5 reductase can improve the resistance of yeast to hydrolysate inhibitors and therefore increase the ethanol production rate on glucose.

On xylose, the much slower sugar consumption rate (as compared to glucose) in one of the best reported pentose fermenting industrial yeasts (Demeke et al. [2013b]) and its derivatives emphasized that anaerobic pentose metabolism still requires further improvement, especially in the presence of lignocellulosic inhibitors. Recent comparisons of metabolic profiles between the glucose and xylose consumption phases of *S. cerevisiae* with (Wang et al. [2014]; Ask et al. [2013]) and without inhibitors (Bergdahl et al. [2012]; Matsushika et al. [2013]), highlighted the significant perturbations in the metabolic capacities of the yeast caused by xylose. In these metabolomics studies, such perturbations were ascribed to the depletion of key metabolites in glycolysis and cofactors (among other important metabolic variations) suggesting inefficient metabolic states such as carbon starvation, and diminished biosynthetic capacities (Bergdahl et al. [2012]; Matsushika et al. [2013]).

Our results also revealed a far more complex set of cellular responses deriving from the interactions between xylose metabolism and *YAP1* overexpression. And although the evaluated physiological responses do not provide enough information for explaining the decrease in xylose consumption in *YAP1*-overexpressing strains, the integration of these observations with the results of previous studies could give some hints for further analysis.

First, higher concentrations of xylitol were obtained for *YAP1*-overexpressing strains, which may result from the unspecific reduction of xylose by the different reductases whose transcription is under the control of *YAP1* (Toone and Jones [1999]) In fact, *YAP1*-overexpressing strains showed a higher xylose reductase activity than the control strain when cells extracts were used for reduction of xylose (data not shown). Possible inhibition of xylose isomerase (XI) by xylitol was considered, although the used XI originates from *C. phytofermentans*, and has been shown to be much less inhibited by xylitol than other previously expressed XIs in *S. cerevisiae* (Brat et al. [2009]). Moreover, the lower xylose consumption for GSE16-YAP1 was observed from the beginning of the fermentation, i.e. when negligible levels of xylitol had been formed. Besides, the rate of consumption was almost constant during most of the process (except at the end of the fermentation when the low concentrations of xylose probably reduce the conversion rate for all strains). This implies that the consumption of xylose did not vary in relation to the increase of xylitol in the medium, i.e. xylitol was not inhibiting the conversion of xylose to xylulose by XI.

A second aspect was connected to the “history” of GSE16. Demeke and co-workers (Demeke et al. [2013b]) reported that the diverse and complex paths followed during the development of GSE16 included unknown mutations that appeared to be linked to the high capacity of the strain to ferment xylose, but that such mutations could also be correlated with a possible detrimental effect in terms of inhibitor tolerance (Demeke et al. [2013b]). The authors suggested that this mutually exclusive phenotype (good xylose fermentation – bad inhibitor tolerance and vice versa) could be either causally or structurally linked, i.e. genes responsible for improved xylose utilization could be functionally connected with genes responsible for the slower growth; or in the other case, such genes may be located close to each other in the genome (Demeke et al. [2013b]). The results obtained in the current study with the *YAP1* overexpressing strains also point towards a mutually exclusive phenotype, but in this case a structural link does not seem likely since the deletion of the locus used for *YAP1* integration (*APJ1*) did not cause a detrimental effect on xylose utilization.

With the exception of xylitol, comparable values were obtained for biomass and extracellular metabolites yields between the different strains in hydrolysate fermentations, which suggested that the overexpression of *YAP1* affected the rate of xylose metabolism but not the product distribution. As similar lower xylose uptake was obtained in mineral medium we could conclude that the negative interactions between xylose metabolism and overexpression of the Yap1 transcription factor were not dependent on the presence of inhibitors and their associated cell responses. When considering available omics data about the effects of overexpressing *YAP1* in *S. cerevisiae* in mineral medium, 17 transcripts were up-regulated by overexpression of the transcription factor (DeRisi et al. [1997]) while around 55 proteins were present in higher concentrations (Jun et al. [2012]). Although the differences in cultivation conditions do not permit to make any conclusion towards particular genes of interest, these two studies suggest that the overexpression of *YAP1* imposes a higher demand to the cells biosynthetic capacity. This, together with an impairment of biosynthetic capabilities on xylose (as presented in the metabolomics studies previously commented (Bergdahl et al. [2012]; Matsushika et al. [2013])), and the high cell maintenance energy required during growth on this sugar (Feng and Zhao [2013]) could explain the slower growth on xylose in *YAP1* overexpressing strains.

Nevertheless, the unexpected results seen with *YAP1*-overexpression during xylose assimilation require deeper analysis to further understand the biological responses that limit the development of robust xylose consuming strains. In this respect, we consider that the study at the molecular level of the cellular responses of *YAP1*-overexpression in GSE16 and other xylose consuming strains (for example expressing the fungal redox pathway) during glucose and xylose utilization would reveal important insights about limiting steps for xylose metabolism.

In conclusion, overexpression of the transcription factor *YAP1* and the mitochondrial reductase *MCR1* in the already robust strain GSE16 resulted in an even faster hexose catabolism in the presence of spruce hydrolysate-derived inhibitors, but the effect was not cumulative. The improved phenotype of *MCR1* overexpression seems to be related, at least in part, to a faster furaldehyde reduction, indicating that this reductase may have a wider substrate range than previously reported. Unexpected reduced xylose fermentation rate was observed in *YAP1* overexpressing strains and further studies are needed to elucidate the mechanisms behind this observation.

## Competing interests

The authors declare that they have no competing interests.

## Authors’ contributions

VWS participated in the design of the study, performed the experiments and wrote the manuscript. LS, YL, MA performed the experiments and commented on the manuscript. MB participated in the initial design of the study and commented the manuscript. DP and PB contributed in conceiving the study and revised the manuscript. MFM and JT participated in design of the study and revised the manuscript. MGG conceived the study and revised the manuscript. All authors read and approved the final manuscript.

## Additional file

## Supplementary Material

Additional file 1: Table S1. Oligonucleotides used in the current work. Shows the sequence of the primers used during the study. **Figure S1.** Carbon dioxide profile of the strains GSE16 (parental), GSE16-YAP1, GSE16-MCR1, GSE16-YAP1-MCR1 and GSE16- ΔΔAPJ1 during anaerobic batch fermentations of spruce hydrolysate (only shown for the first 35 h). The figure shows the carbon dioxide profile of the different strains evaluated in the study during anaerobic batch fermentations of spruce hydrolysate. Click here for file
