# Supplementary material for: Re-assessment of YAP1 and MCR1 contributions to inhibitor tolerance in robust engineered Saccharomyces cerevisiae fermenting undetoxified lignocellulosic hydrolysate
Source: AMB Express. 2014 Jul 22;4:56. doi: 10.1186/s13568-014-0056-5 (PMC4105880; doi:10.1186/s13568-014-0056-5)
Supplement: Additional file 1: Table S1. — Oligonucleotides used in the current work. Shows the sequence of the primers used during the study. Figure S1. Carbon dioxide profile of the strains GSE16 (parental), GSE16-YAP1, GSE16-MCR1, GSE16-YAP1-MCR1 and GSE16- ΔΔAPJ1 during anaerobic batch fermentations of spruce hydrolysate (only shown for the first 35 h). The figure shows the carbon dioxide profile of the different strains evaluated in the study during anaerobic batch fermentations of spruce hydrolysate. [file s13568-014-0056-5-S1.pdf]

Additional file 1

Article title:

Re-assessment of *YAP1* and *MCR1* contributions to inhibitor tolerance in robust engineered *Saccharomyces cerevisiae* fermenting undetoxified lignocellulosic hydrolysate

Journal:

AMB Express

Author names:

Valeria Wallace-Salinas[1+], Lorenzo Signori[2+], Ying-Ying Li[3,4], Magnus Ask[5], Maurizio Bettiga[5], Danilo Porro[2], Johan M Thevelein[3,4], Paola Branduardi[2], María R Foulquié-Moreno[3,4], Marie Gorwa-Grauslund[1*]

Corresponding author:

Marie Gorwa-Grauslund. Applied Microbiology, Department of Chemistry, Lund University, P.O. Box 124, SE-22100 Lund, Sweden.

marie-francoise.gorwa-grauslund@tmb.lth.se

Table S1. Oligonucleotides used during the study

**Name**	**Sequence 5´----3´**
YAP1-F	CTAGTGAATTCATGAGTGTGTCTACCGCCAAG
YAP1-R	TGATGAAGCTTTTAGTTCATATGCTTATTCAAAGCTAATTG
CassFor	CTAGTGCATGCAGTTTATCATTATCAATACTCGCCATTTC
CassRev	TGATGAGATCTGGCCGCAAATTAAAGCCTTCG
GPD-YAP1-F	GCTAGTGACGTCGCATGCGGGAACAAAAGCTGGAGCTCAG
CYC1t-YAP1-R	GTAGACAGCTGGCTAGCGCGCGTAATACGACTCACTATAGG
HR1-F	AGTAGAGACGTCGACTGGAGCATCGAATCTGTAG
HR1-R	AGTAGAGCATGCGTTATCTTGTGTCCACTGGTAGC
HR2-F	AGTAGAGCTAGCGGAGAAGTCATATGACAGTGTG
HR2-R	AGTAGAGCGGCCGCGAATAGCGTATCTTTCCGCATTG
attBP-F	TATTACGCGCGCTAGCGTGGTCGGCTGGAGATCGG
attBP-R	TGACTTCTCCGCTAGCAGCCGTTATGGCGGGCATC
Ver.ins1-F	CTTCAAGTCTCTTCCAGATGGCAGG
Ver.ins1-R	GTGGATGCCAGGAATAAACTGTTCAC
Ver.ins2-F	CCTCGACATCATCTGCCCAGATG
Ver.ins2-R	GTGGTGAACTTCCCTTGCTTCTCTG
MCR1-F	AGACCCATCACATCGCATCAC
MCR1-R	GAATTAGAGCTTAAAGCCAAGCC
YLR446W Lox-F	TTCGAATTTCTTTAATCAAATCATCTATACGCAGGTCGACAACCCTTAAT
Lox-R	TCTAGCACTATAGATCTTCGTAAGCATCACCTAATAACTTCGTATAGC
YLR446W TER-R	GTATGCTGGAGTAATATTTTATCTGCTCTAGCTGGAGGTAGACAAAGAC
YLR446W-F	AATCGGACGTCGTTCAGAACATGACAATTGAAAGCACT

YLR446W R	GTTGCGACGTCTGATGAAATATATTATTGAACTTGGTTGTC
Fw-APJ1-A2	AAGTAAAACACATTTTTCTTTTGCTACCAGTGGACACAAGATA ACGGAGAGTGGTCGGCTGGAGATCGG
Rv-APJ11-A1	AAAAAATAAAAATAGAAAATGTATAACATTACTGCATGACTAT ATAACCTAGCCGTTATGGCGGGCATC
Fw-APJ1-check	GAACGGTAGGTATTGATTGTA
Rv-APJ1-check	AACATATCGGTTCCACCGGT
Fw-A1-attP-tefpr	TTTGTGGTCGGCTGGAGATCGGGTAGTGCCCCAACTGGGGTAA CCTTTGAGTTCTCTCAGTTGGGGGCGTAGAGATCTGTTTAGCTT GCC
Rv-A2-attB-teft	TTTAGCCGTTATGGCGGGCATCGTGGAGTACGCGCCCGGGGAG CCCAAGGGCACGCCCTGGCACCCGCACCGCGGGATGGCGGCGT TAGTATC
Fw-PstI-PhiC31	TTTTCTGCAGGGGTGTCTCGCTACGCCGCT
Rv-BamHI-PhiC31	TTTTGGATCCGGTCGACGAGCTCACTAGTC

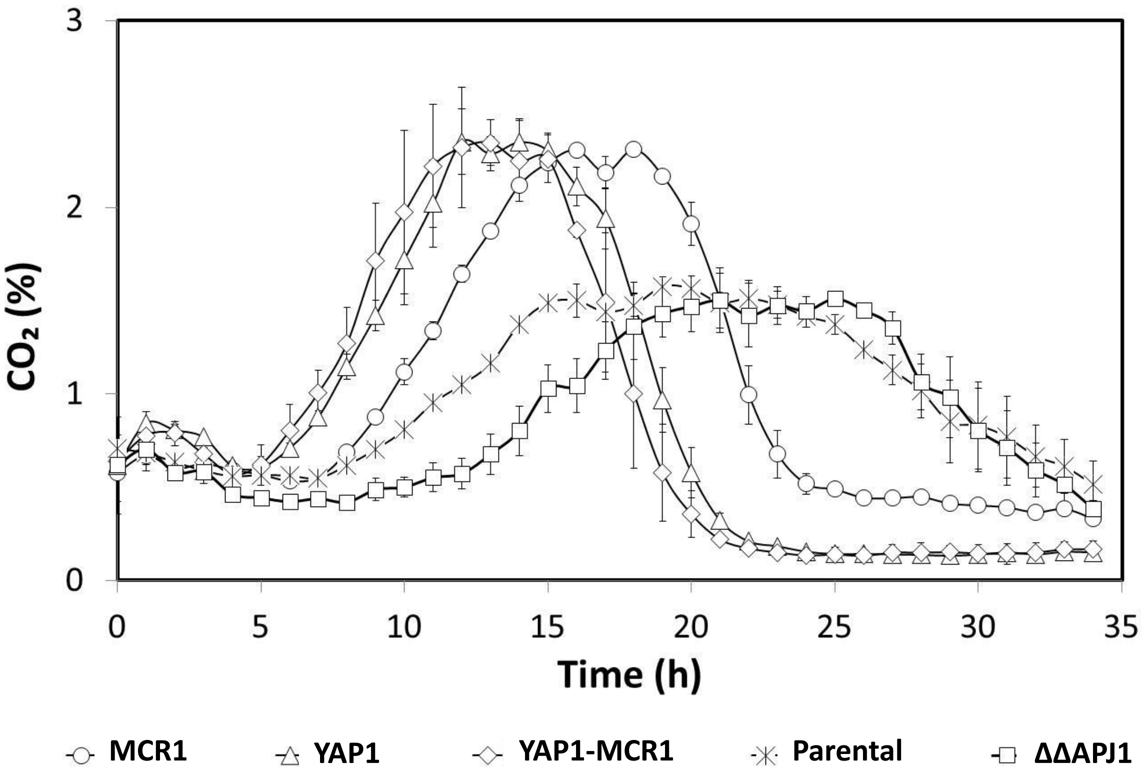

Figure S1. Carbon dioxide profile of the strains GSE16 (parental), GSE16-YAP1, GSE16-MCR1, GSE16-YAP1-MCR1 and GSE16- ΔΔAPJ1 during anaerobic batch fermentations of spruce hydrolysate (only shown for the first 35h)
